# A comparison of the effect of triamcinolone ointment and mouthwash with or without zinc on the healing process of aphthous stomatitis lesions

**DOI:** 10.15171/joddd.2016.014

**Published:** 2016-06-15

**Authors:** Masoumeh Mehdipour, Ali Taghavi Zenooz, Azin Sohrabi, Narges Gholizadeh, Ayla Bahramian, Zahra Jamali

**Affiliations:** ^1^Associate Professor, Department of Oral Medicine, Faculty of Dentistry, Shahid Beheshti University of Medical Sciences, Tehran, Iran; ^2^Associate Professor, Department of Oral Medicine, Faculty of Dentistry, Tabriz University of Medical Sciences, Tabriz, Iran; ^3^Assistant Professor, Department of Pediatric Dentistry, Faculty of Dentistry, Tabriz University of Medical Sciences, Tabriz, Iran; ^4^Assistant Professor, Department of Oral Medicine, Faculty of Dentistry, Tehran University of Medical Sciences,, Tehran, Iran; ^5^Assistant Professor, department of Oral Medicine, Faculty of Dentistry, Tabriz University of Medical Sciences, Tabriz, Iran

**Keywords:** Aphthous stomatitis, zinc mouthwash, triamcinolone with orabase

## Abstract

***Background.*** Recurrent aphthous stomatitis (RAS) is one of the most common ulcerative diseases of the oral mucosa. Definitive etiology of RAS has not been conclusively established. There is no certain treatment for aphthous stomatitis but some drugs such as steroids are commonly used for the treatment of RAS. Regarding the effect of zinc on the healing process of epithelial layer and cell division, in this research the effect of triamcinolone (with orabase) in combination with a zinc-containing mouthwash and triamcinolone alone on the healing process of RAS lesions was assessed.

***Methods.*** The present study consisted of 20 patients diagnosed with RAS. The patients were instructed to rinse the mouth-wash or placebo three times a day and triamcinolone ointment twice a day for two weeks. The largest dimension of the ulcer was measured by a digital caliper and the severity of pain was assessed by visual analogue scale (VAS). Number, size, duration, ulcer-free period and pain of the lesions were evaluated twice a week for twomonths. Data were analyzed by SPSS 16 using Mann-Whitney U test and t-test.

***Results***. A decrease was seen in the mean pain severity score (P = 0.631) and the size of the lesions but it was not statistically significant (P = 0.739). Also the difference between the number of lesions (P = 0.739), duration and ulcer-free period (P = 0.873) were not statistically significant.

***Conclusion.*** Zinc mouthwash seems to be as effective on wound healing process as typical treatment modalities for RAS.

## Introduction

Recurrent aphthous stomatitis (RAS) is one of the most common oral diseases that affects up to 20% of the population.^1,2^ The cause of RAS is unknown, although several factors, including stress, genetics, allergy to certain foods such as milk, cheese and wheat, hematologic disorders, hormonal factors, nutritional deficiencies involving B_12_, folate, zinc, iron etc, are considered as predisposing factors.^1-3^ The mucosal barrier appears to be an important factor in the prevention of aphthous stomatitis and might explain the almost exclusive location of aphthous stomatitis on nonkeratinized mucosa. Numerous factors can disturb the mucosal barrier and increase the frequency of occurrence, including trauma and nutritional abnormalities. Conversely, tobacco use and lutheal phase of the menstrual cycle and pregnancy lead to increased keratinization of the oral mucosa and a decreased frequency of aphthous stomatitis.^2^

The most important factor in the diagnosis of RAS is the history of recurrent ulcers which are induced in regular periods and are usually self-limiting.^4^ Typical RAS lesions are characterized by self-limiting, painful, clearly defined shallow round or oval ulcers, measuring 1‒3 mm, with a shallow necrotic center. A yellow-grayish pseudo-membrane has covered these lesions and are surrounded by minimal raised margins and an erythematous halo.^1^ Despite the use of several drugs such as topical and systemic steroids, Tetracycline, Colchicine, Dapson, Talidomid etc, all of which have side effects, there is no effective treatment for aphthous ulcers. All the treatments are used to reduce pain, healing time, number and size of the lesions, and to prolong ulcer-free periods.^1^

In recent studies on ulcer healing, the effect of trace elements such as zinc has been described. Topical administration of zinc seems to be superior to systemic therapy due to its effect in reducing super-nfections and necrotic material by enhancing local defense systems and collagenolytic activity, and the continuous release of zinc ions which encourage epithelialization of wounds in normozincemic individuals.^5^In 2001 Agren indicated that zinc promotes epithelialization by increasing endogenous growth factors and enzymes and is important for epithelial proliferation and migration.^6^

Due to lack of side effects for zinc gluconate and the efficacy of zinc in epithelial healing, in this study we compared the effect of a zinc-containing mouthwash with that of routine treatment of RAS with topical steroids on the healing process.

## Methods

This double-blind clinical trial was performed in the Faculty of Dentistry, Tabriz University of Medical Sciences. The study protocol was approved by the Ethics Committee of Tabriz University of Medical Sciences (No: 5/4/6380).

Twenty patients with RAS were selected. All the patients signed informed consent forms.

The inclusion criteria consisted of:

- Age over 18 years
- A history of recurrent minor and major aphthous ulcers
- The presence of aphthous ulcers of less than 48 hours of duration

The exclusion criteria consisted of:

- Pregnancy and breast-feeding
- Allergy to zinc or triamcinolone
- Herpetiform aphthous ulcers
- Systemic corticosteroid administration
- Uncontrolled diabetes, gastrointestinal ulcer, or history of tuberculosis
- Drug use for aphthous stomatitis during the previous month
- High blood pressure
- Smoking^7^

After recording the patients’ histories, they were examined by a dentist and the size of the lesions was determined by a digital scale.

Two different mouthwashes called A and B, which were produced in Tabriz Drug Research Center’s laboratory, were utilized in the study. During the study period the observer and the patient did not know what kind of mouthwash (zinc or placebo) was used.

We asked the patients to rinse their mouth with 10 mL of the mouthwash A or B 3 times a day for 1 minute and not to eat or drink anything for 30 minutes after rinsing.

They were asked to apply triamcinolone ointment on oral lesions twice a day and avoid eating or drinking for 15 minutes and then rinse the mouth with water. They used the ointment and mouthwash for 2 weeks.

The patients were examined and followed twice a week for two months and ulcer characteristics, including number (N), size (S), duration (D), ulcer-free period (days) and pain (P) were evaluated in each session.

Pain and discomfort were assessed by 10-point visual analogue scale^1^ and the size of the lesions was evaluated with the use of a digital scale.

Data were statistically analyzed using SPSS 16 with t-test (to compare the two groups) and Mann-Whitney U test (to compare the two groups in each session). Statistical significance was set at P < 0.05.

## Results

In the current study, the severity of the pain, the number of ulcers, ulcer-free period, and the duration and size of the lesions were determined. Severity of pain was measured by VAS criteria. At the initial appointment, the mean score of pain severity was 2.6±0.7 and 2.4±0.8 for the zinc and placebo groups, respectively, which showed no significant differences (P=0.631). Also in the next sixteen sessions no statistically significant differences were seen between the two groups (P=1.00)

**Figure 1:**
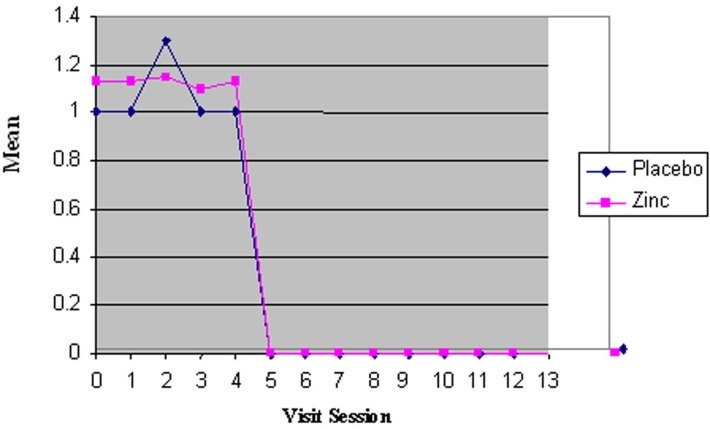
Mean lesion counts in the zinc and placebo groups.

The mean values for the number of lesions in the first session were 1.0±0.0 and 1.1±0.3 for the placebo and zinc groups, respectively, with no significant differences between the two groups (P = 0.739). Also in the next sixteen sessions no statistically significant differences were seen between the two groups (P=0.23; Figure 1).

**Figure 2:**
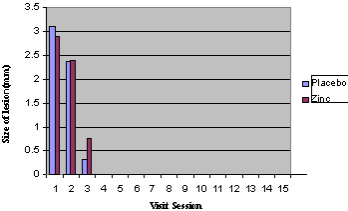
MThe lesion sizes (mm) in the zinc and placebo groups.

At the initial appointment, the mean lesion sizes (the largest length of ulcer) were 2.46±0.44 and 2.58±0.52 for the zinc and placebo groups, respectively, which revealed no significant difference (P = 0.739). Also in the next sixteen sessions no statistically significant differences were seen between the two groups (P = 0.322; Figure 2).

The duration of the lesions was 6.1±1.3 and 6.0±1.4 in the placebo and zinc groups, respectively. T-test and Mann-Whitney test P-values were 0.874 and 0.905, respectively. Lesion-free days during the study were 53.9±1.3 and 53.8±1.5 in the placebo and zinc groups, respectively. T-test and Mann Whitney test P-values were 0.873 and 0.912, respectively. A decrease was seen in the mean of pain severity and the size of the lesions but it was not significant. The differences between the number and duration of the lesions and ulcer-free periods were not significant, either.

## Discussion

As aphthous stomatitis is a recurrent and chronic disease, repeated treatments are necessary. Treatment strategies for RAS should consider the pathogenesis of the disease, therapeutic effects of the drugs, and their side effects.

Several treatment options have been suggested for RAS but due to unknown etiology, most of these treatments are symptomatic and there is no definitive treatment for RAS.^1^

Zinc is an essential trace element in the human body and acts as a cofactor for many metaloenzymes such as alkaline phosphatase, RNA and DNA polymerase, and enzymes which promote debridement and keratinocyte migration in wound healing.^7,8^

Gholizadeh et al^9^ showed a decrease in serum zinc levels in patients with erosive oral lichen planus. Their findings might indicate the promising role of zinc in the development of oral lichen planus.

The value of topical zinc application in wound care has been reported by early observations by Henzel et al.^10^ Their findings showed that in patients following major surgery, a pronounced decline in blood and tissue zinc, together with increased zincuria and loss of zinc in wound exudates/debris resulted in up to a 50% reduction in zinc in the granulation tissue and wound margin, creating a local zinc deficit in patients with poor wound healing.

Henzel et al^10^reported that topical application of zinc is a suitable wound care. They showed that patients undergoing major surgeries and blood loss experienced up to a 50% reduction in zincin margins of the wound and granulation tissue, thus creating local zinc deficit.

Topical zinc administration will lead to epithelial regeneration and endothelium repair. In a double-blind, placebo-controlled trial, zinc oxide promoted healing of ulcers by enhancing local immune system and inflammatory reduction.^6^

Previous studies have showed that zinc administration combined with steroids can reduce symptoms of lichen planus, psoriasis and chronic eczema.^11^

Mehdipour et al showed the effect of 0.2% zinc mouthwash plus fluocinolone on decreasing surface area of oral lichen planus ulcers.^12^

Few studies have been undertaken to assess the effect of zinc on RAS, and most of these studies have evaluated systemic zinc administration.

Orbak et al^7^ assessed the effects of systemic zinc sulfate in the treatment of RAS and after 1 month of zinc therapy, reporting that the aphthae reduced and did not reappear for 3 months. Orbak et al^13^ compared rats with a zinc-deficient diet and rats (controls) with a zinc-containing diet and reported aphthous ulcers in rats with zinc deficiency on the alveolar mucosa with a high rate of 29.9%.

Our results were different from those of Bor et al,^14^ Merchant et al,^15^ Endre et al,^16^ and Orbak et al,^7^ who have shown the efficacy of zinc in treating RAS lesions. This can be due to the route of administration, which was systemic in all these studies.

Our results were also different from those repored by Cummins, who showed that topical zinc citrate in combination with triclosan was effective in the treatment of RAS but this effectiveness can be the result of triclosan administration and not the zinc.^17^

And our results confirmed Wray‘s observations in relation to the effects of systemic zinc sulfate on RAS lesions in 25 patients; after 3 months no changes were detected in 81.3% of the patients.^18^

## Conclusion

Since minor aphthous lesions has a short healing period, we recommend that only patients with major lesions be included in future studies and due to some systemic absorption of topical zinc via mucous and skin, we recommend that topical zinc be assessed in patients with zinc deficiency.

## Acknowledgments

This study was supported by the Research Council of Tabriz University of Medical Sciences. The authors would like to thank the Council for assistance in carrying out this study.

## Authors’ contributions

The study was planned by MM, AT and AZ. AZ and MM carried out the clinical procedures and prepared the drug. The statistical analyses and interpretation of data were carried out by AB. NG, ZJ, and AB were responsible for manuscript preparation. ZJ, MM, and AT critically revised the manuscript for intellectual content. All the authors contributed to the final draft, read and approved the final manuscript.

## Funding

This study was a part of a thesis and research project (Grant No: 1710) supported and funded by Tabriz University of Medical Sciences.

## Competing interests

The authors declare no competing interests with regards to the authorship and/or publication of this article.

## Ethics approval

The study protocol was approved by the Ethics Committee of Tabriz University of Medical Sciences (No: 5/4/6380).
